# Association between generalized joint hypermobility, temporomandibular joint hypertranslation and temporomandibular disorders: a scoping review

**DOI:** 10.1590/1678-7757-2024-0302

**Published:** 2025-03-11

**Authors:** Samilla Pontes BRAGA, Carolina Ortigosa CUNHA, Ambrosina MICHELOTTI, Leonardo Rigoldi BONJARDIM, Paulo César Rodrigues CONTI

**Affiliations:** 1 Universidade de São Paulo Faculdade de Odontologia de Bauru Departamento de Prótese Bauru São Paulo Brasil Universidade de São Paulo, Faculdade de Odontologia de Bauru, Departamento de Prótese, Grupo de Dor Orofacial de Bauru, Bauru, São Paulo, Brasil.; 2 Master Program in TMD/Orofacial Pain Faculty of Dentistry University of Naples Federico II Naples Italy Postgraduate Program in Orthodontics and Master Program in TMD/Orofacial Pain, Faculty of Dentistry, University of Naples Federico II, Naples, Italy.; 3 Universidade de São Paulo Faculdade de Odontologia de Bauru Departamento de Ciências Biológicas Bauru São Paulo Brasil Universidade de São Paulo, Faculdade de Odontologia de Bauru, Departamento de Ciências Biológicas, Seção de Fisiologia da Cabeça e da Face, Bauru, São Paulo, Brasil.

**Keywords:** Temporomandibular joint disorders, Temporomandibular joint, Joint instability, Joint dislocations

## Abstract

Generalized Joint Hypermobility (GJH) is one of the pathophysiological contributing factors for the development of temporomandibular disorders (TMD). There are, however, several counterpoints on the potential relation between TMD and joint hypermobility, especially when considering the temporomandibular joint (TMJ), event known as TMJ hypertranslation. Additionally, there is no consensus regarding the clinical and imaging diagnostic criteria for such condition. Hence, this scoping review addresses the association between GJH, TMJ hypertranslation and TMD, highlighting the lack of consensus concerning TMJ hypertranslation diagnosis. Eligibility criteria included book sections, clinical trials, meta-analyses, multicenter studies, observational studies, and reviews published in English between 1964 and 2024. Bibliographic search was conducted on the PubMed, SciELO, LILACS and Science Direct databases using the following Medical Subjective Headings (MeSH) terms: “temporomandibular joint disorders,” “temporomandibular joint,” “joint instability” and “joint dislocations.” “TMJ hypermobility” and “TMJ subluxation,” non-indexed terms, were applied as individual searches in the same databases. Manual search was performed in selected works by cross-referencing the included studies and book sections. Additional search was conducted in the grey literature. All searches were performed from January to June 2024. After selection, 54 texts were included. While some studies suggest that joint hypermobility (generalized or TMJ specific) may be a risk factor for TMD, especially of the intra-articular type, others rule out this association. No consensus on the potential association between joint hypermobility and TMD was achieved due to the diverse methodologies used to define TMJ hypertranslation diagnosis. More robust and controlled studies are needed to establish a diagnostic criteria and, consequently, understanding of its potential repercussions on masticatory structures, as well as management and prevention of the clinical manifestations.

## Graphical abstract

**Figure:**
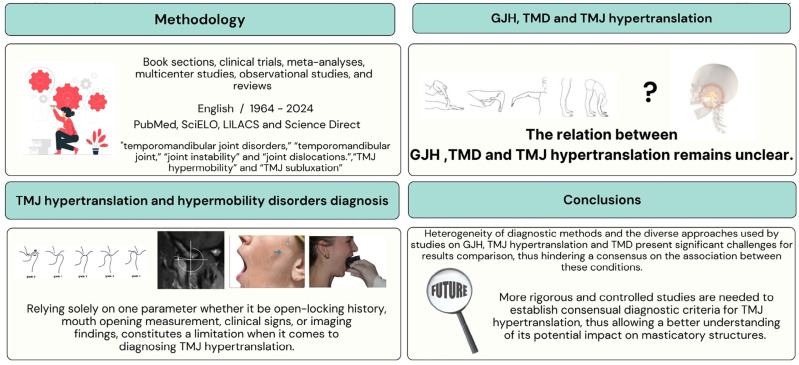

## Introduction

The American Academy of Orofacial Pain (AAOP) defines temporomandibular disorder (TMD) as a collection of conditions affecting the masticatory muscles, the temporomandibular joint (TMJ), and related structures. Although studied for decades, TMD etiology remains a controversial matter. AAOP lists four main factors: trauma, anatomical factors, pathophysiological factors, and psychosocial factors. The pathophysiological factors correspond to systemic features that may act simultaneously at central and peripheral levels. Among these, generalized joint hypermobility (GJH) stands out.^1^

By definition, joint hypermobility refers to excessive movement of a joint within its normal plane. TMJ hypermobility, also known as TMJ hypertranslation, is characterized by joint movement beyond the normal range during condylar translation.^2^ GJH occurs when joint hypermobility affects multiple joints, particularly limbs and axial skeleton.^3^ In the early 1990s, a higher prevalence of GJH in patients with internal TMJ disorders compared to patients with other types of TMD and healthy individuals was reported.^4,5^

Since then, the association between TMD and joint hypermobility, whether generalized or TMJ specific, remains lacking in studies and presents several counterpoints. Similar to disc displacement with reduction (DDWR), TMJ hypertranslation is a clinical condition that continues to raise numerous questions for both patients and clinicians concerning its actual risks, likelihood of progression, and necessity for treatment.^6^

A scoping review approach is ideal for this topic as it maps all relevant aspects of the theme, especially in broad and complex areas like joint hypermobility (both localized and generalized) in which terminology is often inconsistency and definitions of key concepts vary. Based on the research question “What is the evidence on the association between GJH, TMJ hypertranslation and TMD in patients evaluated in clinical settings?” the present scoping review investigates the relation between GJH, TMJ hypertranslation and TMD, focusing on terminology, measurement methods, and risk factors. As a secondary objective, it highlights the lack of consensus on TMJ hypertranslation diagnosis. Importantly, although TMJ hypermobility is used as a synonym for TMJ subluxation and TMJ hypertranslation, the latter will be used throughout the article for reading and understanding purposes.

## Methodology

This study follows the PRISMA guidelines for scoping reviews^7^ and is registered under protocol number 84QHJ on the Open Science Framework Website. The research question was: “What is the evidence on the association between GJH, TMJ hypertranslation and TMD in patients evaluated in clinical settings?” In addition to examining the association between GJH, TMJ hypertranslation and TMD, this review verified the available clinical and imaging methods for diagnosing TMJ hypertranslation.

Bibliographic search was conducted on the PubMed, SciELO, LILACS and Science Direct databases using the following Medical Subjective Headings (MeSH) terms: “temporomandibular joint disorders,” “temporomandibular joint,” “joint instability” and “joint dislocations.” “TMJ hypermobility” and “TMJ subluxation,” non-indexed terms, were applied as individual searches to ensure inclusion of studies that do not use MeSH terminology but are still relevant to the research question.

Search date, database, keywords, applied filters and results are represented in Figure A1 and A2, in Appendix A.

Manual search was performed in selected works by cross-referencing the included studies and consulting books written by experts in the area of orofacial pain or orthopedics to complement the scientific literature, thus ensuring the inclusion of information not widely available. The books were available in the University’s Central Library and in digital collections. Manual search involved consulting the books’ index and summary using terms such as ‘TMJ hypermobility,’ ‘TMJ subluxation,’ and ‘Hypermobility disorders of the TMJ.’ Chapters and related sections were analyzed to identify pertinent information. Books published between 1990 and 2024, in English, with chapters directly related to orofacial pain or joint hypermobility were included. Additional search was conducted in the grey literature using the Opengrey website to ensure that studies not indexed in traditional databases were also appraised. All searches occurred from January to June 2024, updated until June 15, 2024.

Eligibility criteria included book sections, clinical trials (including randomized controlled trials), meta-analyses, multicenter studies, observational studies, and reviews (including systematic reviews) published in English between 1964 and 2024, based on the availability of relevant literature. More specifically, studies about diagnostic criteria for GJH and clinical trials evaluating pain, fatigue, and kinesiophobia. Clinical trials, cross-sectional studies, case-control studies, literature reviews, and meta-analyses investigating the association between joint hypermobility (generalized or TMJ localized) and TMD were included. As were articles and book sections that addressed the study of clinical and imaging signs of TMJ hypertranslation and diagnostic criteria for TMJ hypermobility disorders, such as TMJ subluxation. Clinical studies investigating joint hypermobility (generalized or TMJ localized) treatment and its possible repercussions were also eligible. Extracted variables included participants’ demographic characteristics like age and type of joint hypermobility diagnosis (general or TMJ localized), data on diagnostic criteria for GJH (e.g., the Beighton test) and TMJ hypertranslation diagnosis (anamnestic, clinical, and imaging criteria).

Studies that did not directly address the association between GJH, TMJ hypertranslation, and TMD, or did not focus on these conditions, were excluded. Studies including populations outside the scope of the review, such as children and older adults, were also excluded due to significant differences in clinical manifestations and management of these conditions across different age groups. Additionally, studies with inadequate methodology were excluded, including those lacking sufficient data for result extraction or with selection bias (e.g., unrepresentative samples or flawed sampling strategies).

Critical evaluation of the evidence sources was not performed, as the main objective of this review was to map concepts and identify gaps in research rather than to assess the methodological quality of the studies. Despite no formal assessment of the methodological quality of the studies, the presence of certain limitations was identified and discussed when interpreting the results.

Duplicates were checked using the software Rayyan. Two reviewers selected studies identified through electronic and manual searches by reading the title, and the abstract of relevant studies was read to identify eligibility. In case of disagreement, a consensus between the two reviewers was reached. A standardized form including fields for author name, year of publication, methodology, main results, and conclusions of the study, developed by the authors, was used to extract data from each study. At the end of the analysis, 54 texts were selected. Results were grouped by types of association (e.g., GJH and TMJ hypertranslation in patients with TMD). Qualitative analysis focused on the trends observed in the studies, such as the correlation between GJH and TMD prevalence, and the implications for clinical practice.

Interpretation of the results considered the limitations of each study, focusing on the diagnostic criteria and indicating areas of knowledge gaps that deserve further investigation. The findings and limitations of the studies are described here. The main results of studies relating GJH, TMJ hypertranslation and TMD were summarized in Figure 1, whereas the main results concerning diagnostic criteria for TMJ hypertranslation were summarized in Figure 2.

**Figure 1:** Summary information from studies relating GJH, TMJ hypertranslation and TMD.

Article	Aim	Methodology	Results	Conclusions
Westling L^17^ (1989)	To study the relationship between GJH and TMD	Case-control study with 74 female patients with TMD and 73 healthy controls.	83% of patients with a Beighton score ≥ 3 had TMJ involvement. 41% of patients with a score 0-2 (no joint laxity) also had TMJ involvement. The difference between these two groups was statistically significant.	Patients with more joint hyperlaxity are more likely to have TMD.
Westling L, Mattiasson A.^18^ (1992)	To assess the correlation between joint hypermobility and TMD in adolescents.	Epidemiological study involving 96 girls and 97 boys	2% of girls and 3% of boys had a Beighton score of 5 or more, indicating high joint mobility. Higher joint mobility was correlated with a higher prevalence of TMJ sounds, particularly reciprocal clicking. Adolescents with high joint mobility reported more severe symptoms of TMD, including difficulty in mouth opening and pain during jaw movement. Reciprocal clicking was significantly associated with TMJ tenderness, pain, and joint dysfunction signs like jaw deviation.	GJH, especially in females, was linked to higher occurrences of internal derangement of the TMJ, including reciprocal clicking. This suggests that joint hypermobility may be a contributing factor to TMD.
Ögren, et al.^19^ (2012)	To investigate the role of GJH, TMJ hypermobility and previous jaw trauma in the development of TMJ disc derangement.	Case-control study with 42 patients (21 with RC and 21 with CCL) and 20 control individuals.	Reciprocal clicking (RC) was strongly associated with both GJH and TMJ hypermobility. Chronic closed lock (CCL) was also significantly associated with GJH.	The study suggests that GJH and TMJ hypermobility plays a key role in the onset of TMJ disc disorders, while trauma does not appear to be a significant contributing factor.
Hirsch C, John MT, Stang A.^20^ (2008)	To analyze whether GJH is a risk factor for TMD.	Cross-sectional study with 895 adults	Subjects with four or more hypermobile joints had a higher risk of reproducible TMJ reciprocal clicking. Individuals with GJH had a lower risk of limited mouth opening. No significant association was observed between GJH and myalgia/arthralgia.	GJH may predispose individuals to mechanical TMJ disorders without pain involvement.
Westling L, Mattiasson A.^22^ (1991)	To examine the correlation between TMD symptoms and inherited factors, particularly GJH, as well as the role of oral parafunctions in adolescents.	Cross-sectional study with 193 adolescents (96 girls and 97 boys)	22% of girls and 3% of boys were considered extremely hypermobile (Beighton score > 5). Girls reported significantly more TMD symptoms than boys. GJH appeared to play a key role in the development of TMD when the masticatory system was exposed to local forces, such as oral parafunctions.	Female adolescents may be more predisposed to TMD due to the higher prevalence of GJH in this group.
Conti PC, Miranda JE, Araujo CR.^23^ (2000)	To evaluate the correlation between GJH, TMJ hypertranslation, and signs and symptoms of TMJ intra-articular disorders.	Cross-sectional study with 120 individuals divided into two groups: Symptomatic: 60 patients with complaints of joint noises, pain, or jaw locking. Nonsymptomatic:60 individuals with no TMD complaints.	GJH and Intra-articular Disorders: No significant association was found. GJH and TMJ Hypermobility: No significant correlation was found.	GJH may not be a major factor in the development of TMD.
Chiodelli L, et al.^24^ (2016)	To evaluate dental occlusion and the TMJ in women with and without GJH	Cross-sectional study with 43 women (17 in the hypermobility group and 26 in the non-hypermobility group)	The hypermobility group had higher frequencies of joint noise and deviation during mouth opening, though these differences were not statistically significant. No significant differences were observed between the groups regarding Angle Class.	Hypermobility did not significantly influence dental occlusion or the range of mandibular motion in the women assessed.
Barrera-Mora JM, et al.^25^ (2012)	To investigate the association between malocclusion, GJH, condylar position, and TMD symptoms	Cross-sectional study involving 162 subjects	Significant associations were found between: Skeletal pattern and malocclusion. Transversal malocclusion and malocclusion pattern. Posteroinferior synovial pain and condylar position. Joint hypermobility scale and gender and malocclusion pattern. TMJ function impairment and gender. Sagittal malocclusion pattern and TMD symptoms on the right side. TMJ function impairment and TMD symptoms on both sides. Mandibular motion and TMD symptoms.	There is no statistically significant relationship between GJH and the amount of condylar displacement or TMD.
Davoudi A, et al.^27^ (2015)	To investigate and compare the activity of masticatory muscles in individuals with healthy TMJ and those with mild, moderate, and severe TMJ hypermobility (TMJH).	Clinical study involving 69 patients (aged 22-42) with TMJH and 20 healthy individuals as a control group.	Significant differences in the frequency, duration of activity, and resting periods of masticatory muscles were observed in all TMJH groups (light, moderate, and severe) compared to the healthy group. The severity of TMJH was associated with a reduction in the masticatory muscle activity.	Masticatory muscle activity decreases with the severity of TMJH and excessive mouth opening. The study suggests that TMJH may impair the normal functioning of masticatory muscles, particularly in cases of severe hypermobility.
Dijkstra PU, Kropmans TJ, Stegenga B^26^ (2002)	To analyze the conflicting evidence in the literature regarding the association between TMD and GJH	Literature review and meta-analysis	The odds ratio for hypermobility in TMD cases was 5.4 (26 hypermobile cases and 5 hypermobile controls). In sensitivity analysis, the odds ratio shifted from significant to non-significant in two out of five scenarios	The association between GJH and TMD remains unclear. The study indicates that more rigorous and controlled studies are necessary to draw definitive conclusions.

**Figure 2:** Nomenclature and variables considered for diagnosing TMJ subluxation (or TMJ hypermobility) and TMJ hypertranslation according to each author and year.

Authors	Bell^**30**^ (1990) and Okeson^**29**^ (2019)	Tamimi and Hatcher^**33**^ (2016)	Boering^**34**^ (1966) and Dijkstra^**35**^ (1993)	Diagnostic Criteria for TMD (DC/TMD)^**37**^ (2014)
Nomenclature	TMJ subluxation (or TMJ hypermobility)	TMJ subluxation (or TMJ hypermobility)	TMJ hypertranslation	TMJ subluxation
Clincal examination	At the final stage of opening, the condyle moves forward, creating a small depression in the face behind it. The midline path of mandibular opening shifts away from the center and then realigns as the condyle moves over the eminence.	Wide-open position: The condyle shifts forward beyond the articular eminence, accompanied by an eminence click, incoordination between the bony condyle, disc, and articular eminence, along with deviations of the jaw from the midline.	Nothing to declare	Although diagnostic tests are not necessary, when this condition is clinically present, the physical examination typically reveals the inability to return the mouth to a normal closed position without the patient having to use a manual maneuver.
Imaging findings	Nothing to declare	Open mouth: condyle positioned more than 2-3 mm anteriorly and superiorly to the crest of the eminence.	0 = severe movement limitation(no condyle movement during mouth opening); 1 = restricted movement (condyle positioned posterior to the crest of the eminence during full mouth opening); 2 = normal mobility (condyle positioned at the level of the crest of the eminence during full mouth opening); 3 = tendency toward hypertranslation (condyle positioned anteriorly, but still below the level of the crest of the eminence); 4 = hypertranslation (condyle positioned anteriorly and above the level of the crest of the eminence).	When confirmation is required, imaging findings show the condyle positioned beyond the height of the articular eminence, with the patient unable to close the mouth.
History	Nothing to declare	Nothing to declare	Nothing to declare	Patient's report of being unable to return to a closed position without performing a specific maneuver in the past thirty days.

## Literature review

### Generalized Joint Hypermobility (GJH)

Joint hypermobility is usually asymptomatic for many patients; however, a biomechanical issue related to this factor is that joints within the hypermobile range may be overstrained and more prone to injury from repetitive use. Joint stability primarily relies on the ligaments, muscles, tendons, and joint capsule, as well as the surrounding soft tissues.^8^ Hence, joint hypermobility can occur when one or more of these structures are deficient, leading a hypermobile joint to increasingly rely on the function of muscles and tendons for stability which can result in muscle tension, muscle spasms, tendinitis, and/or pain.^3^

GJH may occur as an isolated condition or as a characteristic of various inherited connective tissue disorders, such as the hypermobile Ehlers-Danlos syndrome (hEDS) which is the most common hereditary connective tissue disorder.^9^ Pain is typically the primary complaint, with joint pain being especially common in weight-bearing joints (hips, knees and ankles), those involved in repetitive movements (shoulders, hands and wrists), as well as the back, neck, and TMJ. When present, pain is often of muscular origin and characterized by tender points at the myotendinous junctions and muscle tension, with or without spasms.^10,11^ Many individuals with joint hypermobility also report fatigue, which is probably the second most common complaint. Additionally, joint pain and dysfunction can contribute to kinesiophobia.^12,13^

GJH is a frequently observed clinical feature of significant importance for treating musculoskeletal conditions, more commonly found in younger patients and is typically linked to a higher incidence of musculoskeletal injuries. It has also been associated with ankle sprains, shoulder instability, anterior cruciate ligament injuries and hand osteoarthritis. GJH can also serve as a direct risk factor for both acute and chronic musculoskeletal injuries, also being linked to chronic pain syndromes and fibromyalgia.^3^ Wolf, Cameron, and Owens^14^ (2011) highlight the necessity for specialized rehabilitation in GJH patients as it can improve proprioception, muscle strength, and balance.

An initial categorization of joint hypermobility was proposed by Carter and Wilkinson^15^ (1964) when they observed capsular laxity in conjunction with congenital hip dislocation. The authors developed tests for joint hypermobility, including hyperextension of the fingers, elbows, knees, and ankles, and compared the results between children with congenital hip dislocation and a counterpart control group. According to the criteria established by these authors, about half of the children with congenital hip dislocation presented joint laxity compared with only 7% of the control group.^15^ Beighton and Horan adapted the Carter-Wilkinson criteria to assess patients with joint laxity and Ehlers-Danlos syndrome, and the Beighton scale has become the gold standard for assessing joint laxity and has been widely used in numerous studies to evaluate the presence and degree of joint hypermobility.^16^

### GJH, TMD and TMJ hypertranslation

Studies indicate that GJH may be a risk or a predisposing factor for TMD development, indicating an increased TMD prevalence in hypermobile individuals compared with controls.^17-19^ In 2008, a cross-sectional assessment of 895 individuals evaluated whether GJH is a risk factor for TMD, showing that individuals with GJH had a higher risk of TMJ noises as a sign of TMJ disc displacement and simultaneously a reduced risk of having limited mouth opening capacity. No correlation was found between GJH and masticatory myalgia and/or TMJ arthralgia, suggesting that GJH may be linked to non-painful TMD subtypes.^20^

Considering TMJ biomechanical principles, it was hypothesized that TMJ hypertranslation, characterized by excessive movement, evolves with stretching of the disc ligaments which, combined with other contributing factors, may lead to joint overload, disc displacement, pain and ‘open-locking,’ the latter characteristic of TMJ subluxation and luxation (hypermobility disorders).^4,21^ Thus, what is known about the impact of TMJ hypertranslation are the TMJ hypermobility disorders.^1^

An important methodological gap observed in research on TMJ hypertranslation is the difficulty of establishing a correct and scientifically accepted diagnosis. Only a few studies in the field of orofacial pain address TMJ hypertranslation, and they use different methods to assess it like mouth opening measuring and imaging exams. Some studies from the 80s and 90s suggested that TMJ hypertranslation, evaluated mainly based on the anterior positioning of the condyle to the articular eminence and TMD signs and symptoms, were linked to GJH.^4,18,22^

In 2000, however, one study evaluated the correlation between GJH, TMJ hypertranslation and signs and symptoms of intra-articular TMJ disorders and found no association between intra-articular TMJ disorders and GJH, neither between GJH and TMJ hypertranslation.^23^ Another study observed that GJH did not influence occlusion and mandibular movement amplitudes in women. Additionally, there was no significant difference in the presence of TMJ noises when comparing hypermobile women and controls.^24^ In 2012, a study found no statistical correlation between GJH and the amount of condylar displacement or TMD.^25^ Importantly, while the first and third studies used images to assess condyle displacement in relation to the articular eminence at maximum opening as a criterion for diagnosing TMJ hypermobility, the second one used clinical parameters such as mouth opening and TMJ noises for this purpose. These studies considered the association between GJH, TMJ hypertranslation and TMD as weak or requiring further investigation. Moreover, the systematic review by Dijkstra, Kropmans, and Stegenga^26^ (2002) concluded that the relation between GJH and TMD remains unclear, and that further rigorous studies are necessary to clarify this issue.

In 2015, one study used the mouth opening measurement to diagnose TMJ hypermobility and collected electromyographic data. It divided patients into three hypermobile groups based on maximum mouth opening (MMO): mild hypermobility (MMO between 50-55 mm); moderate (MMO between 55 and 65 mm); severe hypermobility (MMO over 65 mm), and a control group (MMO < 50 mm). All three hypermobility groups showed significant differences in frequency, activity, and rest time compared with control during chewing and maximum voluntary clenching, suggesting that the activity of the masticatory muscles decreased with TMJ hypermobility severity and greater excessive mouth opening.^27^ One factor that hinders comparing the studies in question is the absence of diagnostic criteria for TMJ hypertranslation: while some articles are based on the degree of condylar displacement, others are based on clinical parameters such as mouth opening measurement or TMJ noises. These studies and their main findings are summarized in Figure 1.

Importantly, several aspects involved in studies on GJH, TMJ hypertranslation and TMD, such as TMD diagnostic method, GJH diagnostic methods and cutoff points, and especially the heterogeneity of diagnostic criteria used for TMJ hypertranslation, are diverse and controversial.

### TMJ hypertranslation and hypermobility disorders diagnosis

According to AAOP, jaw opening is within the normal range when between 40 and 55 mm; however, a mouth opening above 55 mm does not necessarily indicate TMJ hypertranslation. Individual anatomical variations, age, and facial pattern must be considered when judging TMJ mobility.^1,28^ In other words, TMJ hypertranslation diagnosis should not be conducted based only on mouth opening capacity. Other clinical and imaging parameters must be included for a more accurate diagnosis. The history of open lock episodes, difficulty in closing the mouth, and condylar movement beyond the fossa are important factors in establishing TMJ hypertranslation. As the mouth opens fully, some joints may present a brief pause followed by a sudden jump or leap to the maximally open position. This can be easily observed by the physician when examining the patient’s side profile.^29^

From a biomechanical standpoint, TMJ hypertranslation occurs when the disc and the condyle jump forward and upward, in front of the crest of the eminence, followed by a sudden and irregular movement accompanied or not by a joint noise described as a ‘thud.’ This movement can still occur without noises and be clinically noticeable to the examiner from a frontal view during clinical examination, such that the condyles protrude outward (laterally) compared to the rest of the face, characterizing lateral condylar translation. Additionally, both at the end of maximum opening and at the beginning of mouth closure one condyle may precede the other in movement, causing a sudden sideways swing of the jaw that precedes the translation, visible in clinical examination as midline deviation. Both the ‘thud’ noise and the sudden swing of the jaw described can be complaints of patients with TMJ hypertranslation or physical examination findings.^21^

Bell^30^ (1990) and Okeson^29^ (2019) consider TMJ subluxation and TMJ hypermobility as synonyms, where TMJ exhibits a sudden forward movement of the condyle during the final phase of mouth opening. As the condyle moves past the eminence crest, it seems to abruptly shift forward into fully open position; as the condyle moves beyond the crest, it appears to jump forward into wide-open position. Some patients report jaw clicking, but upon clinical examination the click does not resemble that of a disc displacement—the sound is more accurately described as a ‘thud.’ Clinically, this can be observed by asking patients to open their mouth wide. In the final stage of opening, the condyle shifts forward creating a small depression in the face behind it, and the lateral pole may be palpated or seen during this movement. Midline trajectory of the mandibular opening shifts off-center and then returns as the condyle moves over the eminence. The authors also note that this condition is not typically associated with other complaints unless it is accompanied by myogenous pain due to dislocations or when, during mouth closing, the condyle encounters significant difficulty in passing over the eminence crest or the anterior band of the disc, preventing proper mouth closure.

The term symptomatic TMJ hypermobility also appeared as a mild form of subluxation, with patients frequently reporting clicking sounds in the joint and jerky movements of the lower jaw during wide mouth opening and closing.^31,32^ Tamimi and Hatcher^33^ (2016) also consider TMJ subluxation as a synonymous of TMJ hypertranslation, defined as a sudden shift forward of the condyle during the final phase of mouth opening, i.e, the condyle moves past the articular eminence, jumping forward into the full open position. Additionally, condylar subluxation causes an eminence click, lack of coordination between the disc, bony condyle and articular eminence, and shifts of the jaw from the midline. As for imaging as a complementary exam, during mouth opening the condyle is positioned more than 2-3 mm anterior and superior to the crest. We can thus affirm that these authors mainly considered factors related to clinical examination and imaging tests to diagnose TMJ subluxation or hypermobility.

Boering^34^ (1966) and Dijkstra^35^ (1993), from the same study group, proposed a diagnostic criteria for TMJ hypertranslation based on the position of the condyle in relation to the articular eminence, observed in transpharyngeal x-rays during maximal mouth opening. TMJ hypertranslation was classified as: 0 = severe movement limitation (no condyle movement during mouth opening); 1 = restricted movement (condyle positioned posterior to the crest during full mouth opening); 2 = normal mobility (condyle positioned at the level of the crest during full mouth opening); 3 = tendency toward hypertranslation (condyle positioned anteriorly, but still below the level of the crest); 4 = hypertranslation (condyle positioned anteriorly and above the level of the crest).

In fact, a 2014 study positively correlated mouth opening range and the most anterior condyle placement by finding a correlation between higher levels of TMJ hypertranslation (measured by increased mouth opening capacity) with condyle positioning relative to or in front of the articular eminence, especially during wide mouth opening.^36^ Conversely, following the Diagnostic Criteria for Temporomandibular Disorders (DC/TMD), hypermobility disorders can be divided into two types. The first type involves the condyle shifting anteriorly and superiorly past the eminence, and being unable to return to the closed position without a specific maneuver from the patient (subluxation or partial dislocation); the second type requires professional assistance to return the joint to a ‘normal’ condition (luxation or dislocation).^37^ This suggests that for some authors, TMJ subluxation (hypermobility disorders) diagnosis is determined through clinical signs and imaging findings, whereas according to the DC/TMD it is based on the patient’s report of open-lock events.

One can assume that one of the reasons for including TMJ subluxation in the DC/TMD, and the fact that diagnosis is made solely based on patients’ open-lock reports, likely stems from the study by Kalaykova, et al.^31^(2006). In this study, symptomatic TMJ hypertranslation was diagnosed by identifying disruptions in normal mandibular movements, including clicking sounds at the end of mouth opening and/or at the onset of mouth closure, as well as jerky lateral jaw movements. The authors found that not only did participants with symptomatic TMJ hypertranslation show large condylar angles, but nearly half of the healthy control individuals also exhibited this characteristic. This suggests that condyle displacement out of the glenoid fossa, anterosuperiorly to the articular eminence, by itself is insufficient to trigger functional signs of hypermobility. They also argue that functional interferences only arise when a condylar position beyond the eminence is coupled with an unfavorable alignment of the jaw opening and closing muscles.^31^ Notably, however, this study included only nine participants in the hypermobile group and nine in the control group. But despite such limited sample size, its findings are noteworthy since the condylar position alone may not be a reliable predictor of functional signs of hypermobility. Additionally, these limited results may challenge several previous studies that used only the condylar position as a diagnostic criterion for TMJ hypertranslation.

Over forty years ago, Obwegeser, et al.^38^ (1987) investigated the anatomical condyle position in relation to maximum mouth opening. In analyzing 51 healthy patients, none of whom had TMJ dysfunctions or TMJ dislocation, they found an average interincisal distance of 50.7 mm and condyle movement beyond the eminence in most individuals (n=41). No significant correlation was observed between maximal vertical movement and the distance covered by the condyle beyond the crest of the articular eminence during mouth opening. Regarding mouth opening ability, it is well established that individuals can differ significantly in the relative amounts of translation and rotation.^39^ Lewis, Buschang, and Throckmorton^40^ (2001) compared gender differences in mandibular movements and movement ranges during opening and closing, and examined the shape of condylar and incisal opening and closing paths, as well as the relationship between incisor and condylar movements. The notable and significant difference observed in the shapes of the opening and closing pathways of men and women suggests potentially important morphological differences in the articular eminence features. The authors also found no significant correlations between condylar translation and incisor opening or closing movements, which restricts the use of incisor opening as a diagnostic tool for assessing condylar function.

Another study found no significant association between the independent (disc position, condylar position and condylar excursion) and dependent variables (maximum mouth opening, pain and maximum lateral movement), thus concluding that the type of dysfunction and the severity of changes observed in imaging exams were not correlated with pain intensity or the range of mandibular motion.^41^ Thus, the request and, mainly, interpretation of imaging exams must be careful and always based on clinical findings.

A previous biomechanical model of open-locks identified two morphological factors in the masticatory system that could potentially contribute to TMJ dislocations: the angle of the anterior articular eminence and a more forwardly inclined working line of the jaw closing muscles.^42^ From this, the same study group argued that the presence of hypermobility disorders, confirmed through clinical diagnosis, is linked to predicting vulnerability to open-locks assessed by patient-specific musculoskeletal models of the masticatory system. The agreement between the clinical diagnosis of hypermobility disorders and the patient-specific biomechanical predictions was coincidental. Additionally, the model’s predictions overestimated the number of participants susceptible to hypermobility disorders, indicating that morphology alone was insufficient to distinguish between patients and controls.^43^

Pullinger^44^ (2013) found that TMJ osseous anatomical and orthopedic organization holds predictive value for distinguishing between non-affected individuals and those with disc derangement subdiagnoses, as well as among different subdiagnoses. This manifests itself as significant interactions between fossa shape and size variation, condyle–fossa position and ratios of joint space. However, TMJ osseous anatomical models account for only about one-third of the variance or differences. While moderately strong models confirm an association between TMJ osseous organization and function, this connection should not be overstated.^44^

Following the DC/TMD, only episodes of open-locking can be identified, but they must have occurred in the last thirty days to meet the criteria for TMJ subluxation.^37^ Thus, the diagnostic criteria does not enable identifying TMJ hypertranslation beyond the occurrence of open-lock events. Considering that joint hypermobility, by definition, refers to excessive movement of a joint within the normal range of motion, we can infer that cases of TMJ luxation and/or subluxation (as per the DC/TMD) can represent a potential consequence or an unfavorable clinical course of TMJ hypertranslation, given that open-locking episodes may prompt patients to seek healthcare services. But the factors that determine which patients with TMJ hypertranslation are asymptomatic and which will develop TMJ luxation or subluxation are still unclear.

Although some studies have found that in patients with TMJ hypertranslation, more specifically TMJ dislocation and subluxation, the anterior inclination angle of the articular eminence is more accentuated than in controls, others indicate that articular eminence morphology does not affect TMJ anterior dislocation. Thus, the influence of the articular eminence morphology remains an open topic in the occurrence of TMJ open-locking episodes.^43,45^

Due to the various counterpoints and methodological limitations found in the studies conducted so far, especially regarding TMJ hypertranslation diagnosis, establishing or dismissing the relation between TMJ hypertranslation and TMD remains impossible. One hypothesis for such difficulty in researching this topic is the absence of well-established diagnostic criteria to assess TMJ hypertranslation. Clearly, relying solely on one parameter whether it be open-locking history, mouth opening measurement, clinical signs, or imaging findings, constitutes a limitation when it comes to diagnosing TMJ hypertranslation.

As shown in Figure 2, different authors consider different variables for diagnosing TMJ hypertranslation or subluxation, whether clinical manifestations, imaging signs or self-reporting. This suggests that more robust studies are needed to establish a diagnostic criteria considering open-locking history, even if it occurred over thirty days ago, patient complaints including non-painful ones (e.g., self-reported muscle fatigue and joint instability), mouth opening pattern, clinical signs such as click at the end of full mouth opening and at the start of mouth closure, midline deviations during mouth opening and closing, and imaging findings that aim to determine the condyle position relative to the eminence.

### Management

Considering the medical scientific literature on joint hypermobility and the lack of information on TMJ joint hypermobility, several articles in the medical field highlight the importance of rehabilitation programs that include exercises to strengthen and stabilize unstable and hypermobile joints, as surgical joint stabilization procedures are only considered for patients who do not respond to conservative treatment.^46-48^

The therapy applied to patients with TMJ hypertranslation vary depending on the level of impairment of quality of life. In more extreme cases, TMJ hypertranslation can manifest as recurrent TMJ luxation, for which conservative management methods include pain relief using analgesics and manual reduction, whereas long-term chronic recurrent dislocation requires surgical methods.^49^ Moreover, in the absence of randomized studies on surgical techniques, autologous blood injection into the superior joint space and pericapsular tissues combined with intermaxillary fixation appears to be the most scientifically supported treatment for recurrent TMJ luxation at present.^50^

Importantly, reversible and conservative treatments should always be considered as the first line of treatment. As part of self-management, patients with TMJ hypertranslation should learn to limit mouth opening, especially when performing natural functions like biting large foods and yawning. Additionally, exercises to strengthen the masticatory muscles can help reduce the repercussions of hypermobility, despite the lack of randomized clinical studies.^21^ Jaw exercises also have high therapeutic value when it comes to treating temporomandibular disorders.^51,52^ It also corroborates with the scientific literature and current medical practice, which highlights the importance of strengthening and stabilization exercises for treating unstable and hypermobile joints.^53^

Anamnesis and research involving patients with TMJ hypertranslation should also include non-painful symptoms such as muscle fatigue, joint instability, and kinesiophobia, which seem to be present in patients with GJH.^12,13^ Moreover, recent findings suggest that non-painful symptoms may precede TMD pain, highlighting the need for increased scientific focus on non-painful musculoskeletal symptoms in future studies.^54^

## Conclusions

Heterogeneity of diagnostic methods and the diverse approaches used by studies on GJH, TMJ hypertranslation and TMD present significant challenges for results comparison, thus hindering a consensus on the association between these conditions. Considering all the points discussed in this review, it becomes apparent that the parameters used to assess TMJ mobility are varied and always employed in isolation; thus, it is clear that relying on a single diagnostic parameter—whether a history of open locking, mouth opening measurements, clinical signs, or imaging findings—may be a limitation in TMJ hypertranslation diagnosis.

More rigorous and controlled studies are needed to establish consensual diagnostic criteria for TMJ hypertranslation, thus allowing a better understanding of its potential impact on masticatory structures. This, in turn, will enable the study and better understanding of measures to manage and prevent possible TMJ hypertranslation clinical manifestations such as orofacial pain, joint instability, muscle fatigue, kinesiophobia, and open lock episodes.
